# A profile of four patterns of vulnerability to functional decline in older general medicine patients in Victoria, Australia: a cross sectional survey

**DOI:** 10.1186/s12877-016-0323-1

**Published:** 2016-08-05

**Authors:** Lenore Beddoes-Ley, Damien Khaw, Maxine Duke, Mari Botti

**Affiliations:** 1 School of Nursing & Midwifery, Deakin University, Geelong, 3220 Australia; 2 Deakin University-Epworth Hospital Centre for Clinical Nursing Research, Richmond, 3121 Australia; 3 Deakin University-Alfred Hospital Nursing Research Centre, Prahran, 3181 Australia

**Keywords:** Vulnerable elders, Functional decline, Activities of daily living

## Abstract

**Background:**

There are limited published data reporting Australian hospitalized elders’ vulnerability to functional decline to guide best practice interventions. The objectives of this study were to describe the prevalence of vulnerability to functional decline and explore profiles of vulnerability related to the performance of physical activity in a representative group of elders in a single centre in Victoria, Australia.

**Methods:**

A cross-sectional survey of patients aged ≥ 70 years (*Mean age 82.4, SD 7 years*) admitted to a general medical ward of an Australian tertiary-referral metropolitan public hospital from March 2010 to March 2011 (*n* = 526). Patients were screened using the Vulnerable Elders Survey (VES-13). Distinct typologies of physical difficulties were identified using latent class analysis.

**Results:**

Most elders scored ≥3/10 on the VES-13 and were rated vulnerable to functional decline (*n* = 480, 89.5 %). Four distinct classes of physical difficulty were identified: 1) *Elders with higher physical functioning* (*n* = 114, 21.7 %); 2) *Ambulant elders with diminished strength* (*n* = 24, 4.6 %); 3) *Elders with impaired mobility, strength and ability to stoop* (*n* = 267, 50.8 %) and 4) *Elders with extensive physical impairment* (*n* = 121, 23 %) Vulnerable elders were distributed through all classes.

**Conclusions:**

Older general medicine patients in Victoria, Australia, are highly vulnerable to functional decline. We identified four distinct patterns of physical difficulties associated with vulnerability to functional decline that can inform health service planning, delivery and education.

## Background

Hospitalisation is a sentinel event that can precipitate functional decline in older people [1, 2]. Functional decline involves a decrease in the level of independence when performing activities of daily living (ADL), culminating in poor patient outcomes including institutionalisation [3] and death [4]. In a large North American study, 43 % older general medicine patients experienced functional decline on admission to hospital [5] while 64 % declined on hospital admission in an Australian study [6]. Prevention of functional decline begins with recognising patients who are vulnerable to its development [7]. Moreover, clear understanding of the older person’s underlying physical capability is essential to recognise the potential for functional restoration [8].

North American researchers have identified that between 52 and 64 % of hospitalised elders are vulnerable to functional decline on admission to hospital [9, 10]. Grimmer et al. identified 52 % of Australian elders admitted to an Emergency Department as at risk of functional decline [11]. Additional observational studies conducted within Australia have identified older general medicine patients diagnosed with dementia and delirium as more vulnerable to functional decline than those without [12, 13]. Due to a dearth of other descriptive studies, the prevalence of vulnerability to functional decline in older general medicine patients within Australia has not been reported.

Measuring vulnerability to functional decline is important in selecting appropriate participants in research and health service evaluation and in targeting and choosing the most appropriate best practice interventions that minimise its onset [14, 15]. For example, ‘care bundles’ of relevant interventions have been used in other settings to minimise delirium and pain to improve length of stay (LOS) and post-discharge readmission rates [16–18]. This approach could potentially be applied to individual patients based on their patterns of vulnerability to functional decline. At present, however, there are limited reports of the susceptibility to functional decline and characteristics of physical impairment in Australian older general medicine patients. Moreover, no other studies have investigated the presence of patterns in vulnerability of older general medicine patients to functional decline. The objectives of this study were to describe the prevalence of vulnerability to functional decline and explore profiles of vulnerability related to physical activity performance in a representative sample of elders screened on admission to a general medical ward.

## Methods

### Participants and setting

Cross-sectional survey of a convenience sample of 526 of 1380 patients aged 70+ years admitted to a general medical service of a 390 bed tertiary-referral metropolitan public hospital in Victoria, Australia between March 2010 and March 2011. Vulnerability data were collected in order to purposively select a sample of 65 participants for in-depth investigation in an institutional case study designed to evaluate and enhance the management of functional status (FS) in older patients admitted for acute medical care. This involved recruitment of consecutive patients from March to December 2010 and purposive recruitment of patients from January to March 2011. Findings of the institutional case study are not presented in this paper.

The present study was approved by The Alfred hospital (Project 177/07) and Deakin University Human Research Ethics Committees [DUHREC] (Project EC-238-2007). Informed written consent was obtained for the 65 participants of the institutional case study. All screened patients, or their proxies, provided verbal consent to participate in screening and for use and publication of their data in a thesis and journal articles. Patients, or their proxies, were interviewed within 48-hours of admission to the ward in the recruitment process to an institutional case study.

**Fig. 1:**
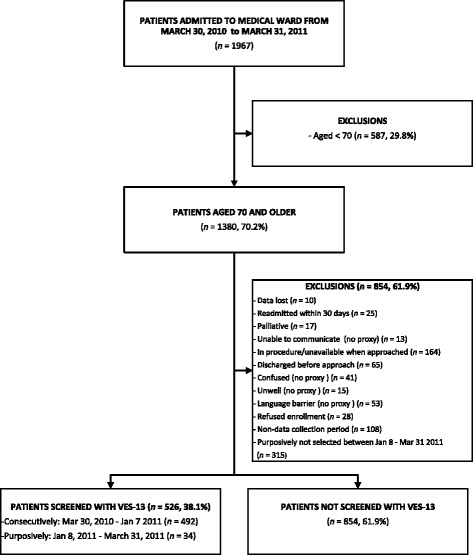
Patient recruitment process in study

Patients were excluded from participating in the survey screen if they were: 1) unable to communicate, with no proxy present; 2) incompetent to provide consent, with no proxy; 3) for palliative (end of life) care; or 4) readmitted to the ward within 30 days of discharge or other episode of acute hospitalisation (see Fig. 1).

The study location was a 32-bed general medical ward staffed by four teams each headed by a general medical consultant and supported by 24-hour nursing care delivery with referral to allied health staff, specialist medical consultants (e.g. geriatricians) and specialist nurses. The Acute-Aged Care Assessment Service provided specialist advice to the treating general medical team regarding the suitability of functionally dependent patients for residential aged care placement on discharge. The model of care delivery emphasised patient access to acute services through efficient treatment of patients’ medical issues. Patients with unresolved functional issues were discharged to rehabilitation programs in sub-acute care. The medical team referred potential candidates for aged care placement on discharge to the Acute-Aged Care Assessment Service for assessment.

### Measures

Sociodemographic data were extracted with permission from the hospital database, including: length of stay, discharge destination, incidence of death, admission diagnosis and International Classification of Diseases (ICD-10) category [19]. Vulnerability to functional decline was evaluated using the Vulnerable Elders Survey (VES-13) [15] as described next.

### The VES-13

The VES-13 is a validated survey used to identify patients at risk of functional decline. It comprises 13 items that assess age, self-rated health, and difficulty performing 6 physical activities and five functional activities of daily living (ADLs) to give a score from 0 to 10 [15]. The VES-13 survey tool scoring process acknowledges an association between increasing age and vulnerability to functional decline according to age group categories [15]. Respondents under the age of 74 years do not attract an age-related score, whereas those aged 75–84 years are assigned one point and those aged 85 years and over score 3 points according to the VES-13 survey tool. Patients were asked to report how they felt and functioned 2-weeks prior to admission to hospital. They were classified ‘vulnerable elders’ with a score of 3 or above. A cut-off score of 3 on the VES-13 had 72.7 % sensitivity and 85.7 % specificity for Comprehensive Geriatric Assessment deficits and was highly predictive for identifying impairment (area under the receiver operating curve, 0.90) [20]. The VES-13 can be completed by self and proxy report [15, 21], and is reliable (Pearson correlation coefficient = 0.92) [20].

### Analysis

To establish the representativeness of screened patients to the total ward throughput of elders aged 70+ years we compared their sex, age, admission diagnosis, length of stay, discharge destination and incidence of death in hospital. Admission diagnosis was categorised according to the International Classification of Diseases (ICD-10) [19]. Length of stay denotes the number of days between admission to, and discharge from, all services within the hospital. We described vulnerability to functional decline in three age groups (70–74 years, 75–84 years, 85+ years) using descriptive statistics. Age was analysed as a categorical variable to reflect increases in the risk of vulnerability as defined in the VES-13 [15].

The potential contribution of VES-13 items to overall vulnerability was reported as dichotomous variables that reflected item thresholds for scoring the VES-13. If elders were ‘unable to do’ or identified having ‘a lot of difficulty’ performing a *physical* activity they were defined as having substantial difficulty, reflecting increased vulnerability to decline. To reflect scoring rules for items measuring difficulties with *functional* activities, elders who reported difficulty performing a functional activity, but received help for it, or avoided performing a functional activity due to health were defined as having substantial difficulty.

All analyses were undertaken using SPSS version 23. We ran non-parametric and parametric statistical analyses according to the level of data (discrete, continuous) and distribution of data (non-normal, normal) to explore associations and differences between patient age group and responses to VES-13 items. Between-group differences in the number of difficulties with physical and functional activities were analysed via one-way Welch’s ANOVA and ANOVA, respectively. Binary logistic regression was used to test associations between age group and the presence of increased vulnerability due to: self-rated health; difficulties with physical activities; and, difficulties with functional activities.

We used MPLUS 5.3 to identify distinct typologies of physical difficulties using latent class analysis (LCA). Dichotomous variables measuring the potential contribution to total vulnerability due to difficulty with physical activities were included in the model. These were the presence or absence of substantial difficulty: (1) *stooping, crouching or kneeling*, (2) *lifting, or carrying objects as heavy as 10 lb*, (3) *reaching or extending arms above shoulder level*, (4) *writing, or handling and grasping small objects*, (5) *walking a quarter of a mile,* (6) *heavy housework such as scrubbing floors or washing windows*. We performed LCA iteratively to identify one through five latent classes. Best model fit was established via parametric bootstrapped likelihood ratio tests of k-1 classes [22].

## Results

### Patient characteristics

Characteristics of all patients on the ward aged 70 years and over are described in Table 1. To test the generalisability of the screening sample to all elders on the ward (*n* = 1380) we compared the demographic characteristics of screened (*n* = 526) and unscreened (*n* = 854) patients. No significant differences were found for age (t (1378) = .152, *p* = .879) or the distribution of sex (*χ*^2^ = 0.042, *p* = .837, with continuity correction), admission reason (Fisher’s exact = 17.216, *p* = .414) or discharge destination (Fisher’s exact = 10.223 *p* = .092) between screened and unscreened elders. However, screened patients had a significantly longer hospital stay (U = 167561.5, *p* < .001, *r* = −.21) and were significantly less likely to die in hospital (*χ*^2^ = 7.797, df = 1, *p* = .005, phi = −.078, with continuity correction) compared with those not screened. These differences are likely to be artefacts of the exclusion of palliative, unwell or confused patients with no proxy present, and, although statistically significant, do not reflect divergence in the core characteristics between groups.

**Table 1:** Characteristics of patients aged 70+ years screened and not screened with the VES-13

Variable	Screened patients (*n* = 526)	Not screened (*n* = 854)	All patients aged 70+ years (*n* = 1380)
Age: Mean (SD)	82.4 (7)	82.3 (7)	82.4 (7)
Sex (*n*, %)			
Male	244 (46.4)	390 (45.7)	634 (45.9)
Female	282 (53.6)	464 (54.3)	746 (54.1)
Length of hospital stay (days)			
Median (IQR)	8 (7)	5 (6)	6 (7)
Min, Max	1, 59	1, 69	1, 69
Discharged (total n, total %)	504 (95.8)	784 (91.8)	1288 (93.3)
Home (*n*, %)	262 (52)	447 (57)	709 (55)
Other hospital (*n*, %)	201 (39.9)	290 (37)	491 (38.1)
Private Hospital (*n*, %)	22 (4.4)	23 (2.9)	45 (3.5)
Residential Aged Care (*n*, %)	6 (1.2)	12 (1.5)	18 (1.4)
Transition care (*n*, %)	11 (2.2)	7 (0.9)	18 (1.4)
Discharged at own risk/absconded (*n*, %)	2 (0.4)	5 (0.6)	7 (0.5)
Died in hospital (*n*, %)	22 (4.4)	70 (8.2)	92 (7.1)
Admission ICD-10 diagnosis (*n*, %)			
I: Infectious and parasitic diseases (A00–B99)	56 (10.6)	73 (8.5)	129 (9.3)
II: Neoplasms (C00–D48)	25 (4.8)	41 (4.8)	66 (4.8)
III: Diseases of the blood and blood-forming organs and the immune mechanism (D50–D89)	9 (1.7)	15 (1.8)	24 (1.7)
IV Endocrine, nutritional and metabolic diseases (E00–E90)	17 (3.2)	31 (3.6)	48 (3.5)
V: Mental and behavioural disorders (F00–F99)	18 (3.4)	35 (4.1)	53 (3.8)
VI: Nervous system diseases (G00–G99)	6 (1.1)	26 (3)	32 (2.3)
VII: Eye and adnexa diseases (H00–H59)	1 (0.2)	1 (0.1)	2 (0.1)
VIII: Ear and mastoid process diseases (H60–H95)	1 (0.2)	2 (0.2)	3 (0.2)
IX: Circulatory system diseases (I00–I99)	123 (23.4)	199 (23.3)	322 (23.3)
X: Respiratory system diseases (J00–J99)	77 (14.6)	116 (13.6)	193 (14)
XI: Digestive system diseases (K00–K93)	25 (4.8)	50 (5.9)	75 (5.4)
XII: Skin and subcutaneous tissue diseases (L00–L99)	12 (2.3)	18 (2.1)	30 (2.2)
XIII: Musculoskeletal system and connective tissue diseases (M00–M99)	28 (5.3)	36 (4.2)	64 (4.6)
XIV: Genitourinary system diseases (N00–N99)	31 (5.9)	60 (7)	91 (6.6)
XVII: Congenital malformations, deformations and chromosomal abnormalities (Q00–Q99)	1 (0.2)	0 (0)	1 (0.1)
XVIII: Symptoms, signs and abnormal clinical and laboratory findings, not elsewhere classified (R00–R99)	26 (4.9)	60 (7)	86 (6.2)
XIX: Injury, poisoning and other consequences of external causes (S00–T98)	68 (12.9)	91 (10.7)	159 (11.5)
XXI: Factors influencing health status and contact with health services (Z00–Z99)	1 (0.2)	0 (0)	1 (0.1)
Missing	1 (0.2)	0 (0)	1 (0.1)

### Profile of vulnerability to functional decline

#### Overall vulnerability and self-rated health

Table 2 reports the vulnerability status and self-rated health of screened patients. Most screened patients scored ≥3/10 on the VES-13 and were rated vulnerable to functional decline (*n* = 471, 89.5 %). While prevalence of vulnerability was high across all age groups the VES-13 automatically defines all elders aged 85 years or over as vulnerable because of their advanced age. To investigate VES-13 vulnerability without the contribution of scores from patient age alone, we deducted scores due to age from participants’ total VES-13 score. Adjusted VES-13 scores indicated that the majority of elders were vulnerable to functional decline for reasons other than age alone (*n* = 431, 81.9 %). This included most patients aged 85+ years (*n* = 182, 87.9 %).

**Table 2:** Overall VES-13 vulnerability and self-rated health in screened patients aged 70+ years

VES criteria	Total patients screened *n* (%)	Aged 70–74 years *n* (%)	Aged 75–84 years *n* (%)	Aged ≥85 years *n* (%)
Total VES-13 score				
Vulnerable (VES ≥3/10)	471 (89.5)	59 (71.1)	205 (86.9)	207 (100)
Not vulnerable (VES <3/10)	55 (10.5)	24 (28.9)	31 (13.1)	0 (0)
Adjusted VES-13 score				
Vulnerable (VES ≥3/10)	431 (81.9)	59 (71.1)	190 (80.5)	182 (87.9)
Not vulnerable (VES <3/10)	95 (18.1)	24 (28.9)	46 (19.5)	25 (12.1)
Self-rated Health				
Poor/Fair	304 (56.7)	52 (62.7)	148 (62.7)	104 (48.8)
Good/Very good/Excellent	222 (42.2)	31 (37.3)	88 (37.3)	103 (49.8)

Over half the sample rated their health as ‘poor’ or ‘fair’ (*n* = 304, 56.7 %). Investigation of frequencies indicated that despite their more advanced age, a lower proportion of elders aged 85 years or over, perceived their health as poor or fair compared to elders in other age groups (see Table 3). Binary logistic regression revealed a statistically significant effect of age group on the likelihood of rating health as poor or fair (*χ*^2^ = 7.964, df = 2, *p* = .019). Elders aged between 70 and 74 years (OR = 1.67, 95 % CI = 0.99–2.8) and 75 to 84 years (OR = 1.67, 95 % CI = 1.1–2.4) had over one and a half the odds of rating their health ‘poor’ or ‘fair’ compared with elders aged 85 years or over.

**Table 3:** Logistic regression: Associations between age group and substantial difficulty with physical activities

	Age group		
Substantial difficulty	70–74 years	75–84 years	85+ years
Stooping, crouching or kneeling			
*n* (%)	54 (65.1)	152 (64.4)	147 (71)
OR	n.a.	0.97	1.31
95 % CI	n.a.	(0.58–1.64)	(0.77–2.26)
Lifting or carrying objects as heavy as 10 lb (4.5 kg)			
*n* (%)	34 (41)	121 (51.3)	127 (61.4)
OR	n.a.	1.51	2.29*
95 % CI	n.a.	(0.91–2.52)	**(1.36–3.85)**
Reaching or extending arms above shoulder level			
*n *(%)	18 (21.7)	58 (24.6)	53 (25.6)
OR	n.a.	1.18	1.24
95 % CI	n.a.	(0.65–2.15)	(0.68–2.28)
Writing or grasping small objects			
*n *(%)	12 (14.5)	35 (14.8)	35 (16.9)
OR	n.a.	1.05	1.22
95 % CI	n.a.	(0.51–2.13)	(0.60–2.48)
Walking a quarter of a mile (400 m)			
*n* (%)	44 (53)	151 (64)	127 (61.4)
OR	n.a.	1.59	1.42
95 % CI	n.a.	(0.95–2.65)	(0.85–2.39)
Heavy housework such as scrubbing floors or washing windows			
*n* (%)	55 (66.3	177 (75)	177 (85.5)
OR	n.a.	1.50	3.1**
95 % CI	n.a.	(0.87–2.59)	**(1.69–5.71)**

Bolding denotes presence of a statistically significant effect (*p* < .05); *p < .005; **p < .001; n.a. not applicable, reference group

#### Self-reported difficulties with physical activities

Most elders reported having at least one substantial difficulty with a physical activity (*n* = 471, 89.5 %). Prevalence of substantial difficulty with physical activities were: stooping, crouching or kneeling (*n* = 353, 67.1 %); lifting or carrying objects as heavy as 10 lb, 4.5 kg (*n* = 282, 53.6 %); reaching or extending arms above shoulder level (*n* = 129, 24.5 %), writing or grasping small objects (*n* = 82, 15.6 %), walking a quarter of a mile, 400 m (*n* = 322, 61.2 %), heavy housework such as scrubbing floors or washing windows (*n* = 409, 77.8 %). A one-way Welch’s ANOVA indicated a small, but statistically significant effect of age group on the number of substantial physical difficulties reported by elders (F (2, 200.873) = 4.313, *p* = .015, η^2^ = .02). Post hoc analysis with the Games-Howell test indicated that patients aged 85+ years (μ = 3.22, SD = 1.59) reported a greater number of substantial physical difficulties than patients aged 70–74 years (μ = 2.57, SD = 1.89, *p* < .05) but not 75–84 years (μ = 2.9, SD = 1.73). There was no significant difference in number of substantial physical difficulties reported between patients aged 70–74 years and patients aged 75–84 years.

Binary logistic regression identified age group as a significant predictor of reporting substantial difficulty lifting or carrying objects as heavy as 10 lb and performing heavy housework (see Table 3). The overall statistical significance for logistic regression models was *p* = .004 (*χ*^2^ = 10.896, df = 2) and p < .001 (*χ*^2^ = 15.366, df = 2), respectively. The odds of elders aged 85 years or over reporting substantial difficulty lifting or carrying objects were approximately twice those of elders aged 70 to 74 years. Furthermore, elders aged 85 years or over had approximately three times the odds of reporting substantial difficulty performing heavy housework compared to those of elders aged between 70 and 74 years.

#### Self-reported difficulties with functional activities

Overall, 411 (78.1 %) screened elders reported having substantial difficulty with at least one functional activity measured by the VES-13. These participants indicated that they had difficulty performing a functional activity, but received help for it, or did not perform a functional activity because of their health. Prevalence of substantial difficulties with functional activities among screened elders was: walking across a room (*n* = 211, 40.1 %); bathing or showering (*n* = 287, 54.6 %); shopping for personal items (*n* = 306, 58.2 %); managing money (*n* = 211, 40.1 %); and performing light housework (*n* = 201, 38.2 %). A one-way ANOVA indicated a small, but statistically significant effect of age group on the number of substantial functional difficulties reported by elders (F (2, 495 = 7.351, *p* = .001, η^2^ = .03). Post-hoc analysis with Tukey’s HSD tests indicated that patients aged 85 years or older (μ = 2.6, SD = 1.7) had a significantly greater number of substantial difficulties with functional activities compared with patients aged 70 to 74 years (μ = 1.9, SD = 1.7; *p* = .006) and patients aged 75 to 84 years (μ = 2.1, SD = 1.7). The number of substantial difficulties with functional activities did not significantly differ between patients aged 70 to 74 years and patients aged 75 to 84 years (*p* = .754).

Binary logistic regression revealed that age group was significantly predictive of having substantial difficulty bathing or showering, shopping for personal items and managing money (see Table 4). The overall statistical significance for these logistic regression models was *p* = .016 for bathing or showering (*χ*^2^ = 8.282, df = 2) and *p* = .001 for shopping for personal items (*χ*^2^ = 14.617, df = 2) and managing money (*χ*^2^ = 14.946, df = 2). Compared to the odds among elders aged 70 to 74 years, elders aged 85 or over had approximately twice the odds of reporting substantial difficulty with these functional activities compared to patients aged 70 to 74 years.

**Table 4:** Logistic regression: Associations between age group and substantial difficulty with functional activities

	Age group		
Substantial difficulty	70–74 years	75–84 years	85+ years
Walking across the room			
n (%)	31 (37.3)	88 (37.3)	92 (44.4)
OR	n.a.	0.96	1.29
95 % CI	n.a.	(0.57–1.61)	(0.76–2.18)
Bathing or showering			
*n* (%)	34 (41)	128 (54.2)	125 (60.4)
OR	n.a.	1.64	2.13*
95 % CI		(0.98–2.73)	**(1.27–3.60)**
Shopping for personal items			
*n* (%)	40 (48.2)	125 (53)	141 (68.1)
OR	n.a.	1.21	2.30*
95 % CI	n.a.	(0.73–2.00)	**(1.37–3.86)**
Managing money			
*n* (%)	27 (32.5)	80 (33.9)	104 (50.2)
OR	n.a.	1.06	2.12**
95 % CI	n.a.	(0.625–1.81)	**(1.24–3.61)**
Light housework			
*n* (%)	32 (38.6)	83 (35.2)	86 (41.5)
OR	n.a.	0.85	1.12
95 % CI	n.a.	(0.51–1.42)	(0.66–1.89)

Bolding denotes presence of a statistically significant effect (*p* < .05); *p < .005; **p < .01; n.a. not applicable, reference group

#### Classes of difficulty with physical activities

**Fig. 2:**
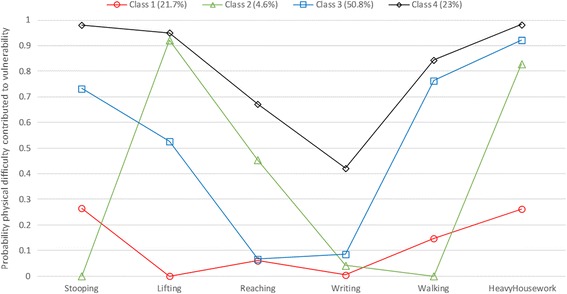
Results of LCA (4-class model) in vulnerable general medical patients (70+ years)

Latent class analysis indicated four distinct classes of difficulty with physical activities (see Fig. 2). First, parametric bootstrapped likelihood ratio tests of k-1 latent classes indicated that the four-class latent class model had significantly greater fit compared to the three class model (*p =* .04), but not the five class model, compared to the four class model (*p* = .286). Moreover, AIC scores were lowest in the four class model (AIC = 3242.972) compared to the three class (AIC = 3247.139) and five class (AIC = 3245.59) model. This confirmed that the four class model had superior fit. We examined the fit of the bivariate items entered into the model with Pearson’s and likelihood Chi-square tests and item responses did not differ from that expected by the model (Pearson’s *χ*^2^: *p* = .571, Likelihood *χ*^2^: *p* = .779). Standardised residuals of bivariate Chi-square tests indicated that variables were independent of one another within latent classes (z_res_ <1.96), thus satisfying the assumption of conditional independence.

Elders in Class 1 (*n* = 114, 21.7 %) had the least likelihood of substantial difficulty across the six physical activities and were labelled *Elders with higher physical functioning* (*n* = 114, 21.3 %). Members of Class 1 possessed lower likelihood of substantial difficulty stooping (26.5 %), walking (14.7 %) and ability to perform heavy housework (26.2 %) and they had no substantial difficulty lifting. Elders in Class 2 (*n* = 24, 4.6 %) had an absence of substantial difficulty stooping and walking, however, they were very likely to have substantial difficulty lifting (92.2 %) and performing heavy housework (82.9 %). Consequently, Class 2 were categorised as: *Ambulant elders with diminished physical strength*. Elders in Class 3 comprised half the sample (*n* = 267, 50.8 %) and were categorised as *Elders with impaired mobility, strength and ability to stoop*. Class 3 elders had high likelihood of substantial difficulty with stooping (73.2 %), walking (76.3 %) and performing heavy housework (92.2 %). These elders also had very low likelihood of substantial difficulty reaching (6.6 %) and writing (8.5 %). Elders in Class 4 (*n* = 121, 23 %) had the greatest likelihood of substantial difficulty with all six physical activities including greater likelihood of substantial difficulty reaching (67.2 %) and writing (42.1 %). These elders were categorised as *Elders with extensive physical impairment who may have difficulty even reaching for or handling objects.*

**Fig. 3:**
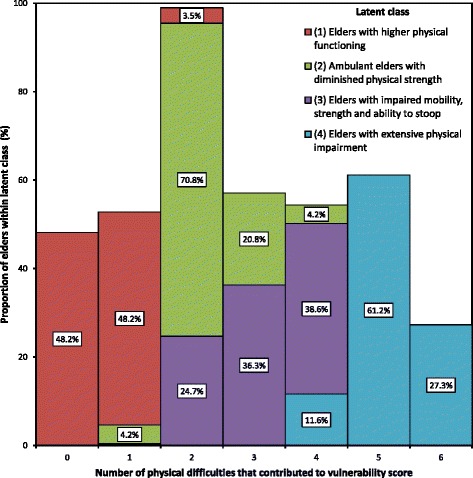
Number of physical difficulties among latent classes of vulnerable elders (70+ years)

**Fig. 4:**
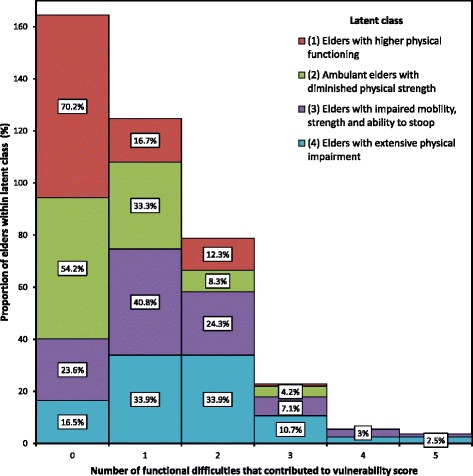
Number of functional difficulties among latent classes of vulnerable elders aged 70+ years

Histograms reporting frequencies of substantial difficulty with physical and functional activities across latent classes are presented in Figs. 3 and 4, respectively. There were distinct differences between latent classes in the number of substantial difficulties reported for physical, but not functional activities. The mean number of substantial difficulties with physical activities increased incrementally through the latent classes: *Elders with higher physical functioning* (Class 1; μ = 0.55, SD = 0.57), *Ambulant elders with diminished physical strength* (Class 2; μ = 2.25, SD = 0.61), *Elders with impaired mobility, strength and ability to stoop* (Class 3; μ = 3.13, SD = 0.8) and *Elders with extensive physical impairment* (Class 4; μ = 5.16, SD = 0.606). The same pattern occurred for the number of substantially impaired functional activities: Class 1 (μ = 0.71, SD = 1.1); Class 2 (μ = 1.63, SD = 1.44); Class 3 (μ = 2.5, SD = 1.6) and Class 4 (μ = 3.6, SD = 1.3).

Chi-square tests of independence indicated statistically significant associations between latent class membership and vulnerability status (see Table 5). *Elders with extensive physical impairment* (Class 4) were significantly more likely than expected to be rated as vulnerable to functional decline (z_res_ = 4.3). Conversely, *elders with higher physical functioning* (Class 1) were less likely than expected to be rated as vulnerable (z_res_ = −4). A one-way Welch’s ANOVA indicated a significant difference in total VES-13 scores between latent classes F (3, 91.658) = 99, *p* < .001, η^2^ = .47). Total vulnerability scores were high in all classes except Class 1. Post-hoc analyses with the Games-Howell test indicated that *Elders with higher physical functioning* (Class1; μ = 3.5, SD = 2.7) had significantly lower VES-13 vulnerability scores than elders in latent class 2 (μ = 7.6, SD = 2.2; *p* < .001), class 3 (μ = 7.7, SD = 1.8; *p* < .001) and class 4 (μ = 8.4, SD = 1.3; *p* < .001). Furthermore, e*lders with extensive physical impairment* (Class 4) had significantly greater VES-13 vulnerability scores than *elders with impaired mobility, strength and ability to stoop* (Class 3; *p* < .001).

**Table 5:** Vulnerability (VES-13) status of medical patients aged 70+ years according to latent class

VES criteria	Class 1 elders with higher physical functioning (*n*, %)	Class 2 ambulant elders with diminished physical strength (*n*, %)	Class 3 impaired mobility, strength and ability to stoop (*n*, %)	Class 4: extensive physical impairment - may have difficulty even reaching for or handling objects (*n*, %)	*X* ^2^ (p)	Cramer’s V
Total VES-13 score					192.514 (<.001)	.605
Vulnerable (VES ≥3/10)	62 (54.4)	23 (95.8)	265 (99.3)	121 (100)		
Not vulnerable (VES <3/10)	52 (45.6)	1 (4.2)	2 (0.7)	0 (0)		

## Discussion

In this study, we found a much higher rate of vulnerability to functional decline than previously reported [9, 10] with 89.5 % of participants rated vulnerable to functional decline 2-weeks preadmission to hospital. Our study findings show that a high proportion of patients (70.2 %) admitted for general medical care were aged 70 years and over. This appears to suggest that patients with general medical conditions were sequestered to the general medicine service on the basis of age alone, thus accounting for the high prevalence of vulnerability. However, our analyses revealed that high vulnerability occurred irrespective of age group or age score adjustment, with the majority of elders (*n* = 431, 81.9 %) rated as vulnerable to functional decline despite their age. This may be associated with the high level of pre-hospital functional decline in Australian older general medicine patients (64 %) identified in previous research [6]. Higher vulnerability in Australian general medicine patients may also be associated with health system factors such as universal access to healthcare provided by the Australian Medicare system, that funds supports for elders to live in the community, thereby influencing patterns of hospitalisation [13].

Clinically and cost effective acute models of geriatric care are available [23, 24] and where operational, we suggest criteria to exclude elders from admission that are more sensitive than age and based on vulnerability should inform patient selection. For example, we found 61.2 % of elders reported difficulty walking one-quarter of a mile, which is associated with greater mortality, new functional disability and additional hospitalisations [25]. Similarly, while self-rated health was associated with greater vulnerability, we found fewer patients (48.8 %) aged 85+ years rated their health poorly than those aged 70–84 years (62.7 %) suggesting in practice that older patients report better health despite being vulnerable to functional decline. Moreover, highest difficulty performing ADLs was identified in those aged 85 years and over. High vulnerability levels in older general medicine patients support the need for good clinical management to minimise functional decline and promote in-hospital recovery [6].

The second major finding was that participants’ preexisting difficulties performing physical activities were distributed into 4 distinct profiles of vulnerability (see Fig. 2). This indicates that to maximise the delivery of safe and quality care, elders should be targeted for best practice multidisciplinary interventions that promote their functional recovery and restoration [26]. Current approaches used in critical care contexts include ‘bundles of care’ strategies to suit the specific needs of older patients to minimise delirium, pain and functional decline [16–18]. Additionally, function-focused care (FFC), also called ‘restorative care’, where nurses help patients engage in the care activity rather than performing the task for them [27, 28] can be individualised according to specific patient needs.

Future ability to identify new patients within classes of vulnerability to functional decline is needed. For example, *ambulant elders with diminished physical strength* had particular difficulty with activities requiring upper body strength. Lean body mass is reduced in age and fat is redistributed [29] increasing the risk of falls in older people [30]. These patients would likely benefit from muscle strengthening interventions that reduce falls risk. Conversely, *elders with higher physical functioning* (Class1; *n* = 114, 21.3 %) had an absence of substantial difficulty lifting. These elders would likely respond best to strategies that promote independence in hospital*.* For example, evaluation of mobility programs have indicated that daily care that incorporates 2 to 4 walks per day of approximately 10 to 20 min duration can improve functional outcomes in general medicine patients who are capable of ambulating [31–36]. Moreover, a recent randomized controlled trial indicated that compared to controls, hospitalised elders at high risk of readmission who were capable of participating in a tailored exercise program and multidisciplinary follow up care had a significant improvement in their abilities to perform instrumental ADLs and walking [26].

Our findings demonstrate that older general medicine patients were likely to require high levels of support to mobilise and complete personal and instrumental ADLs safely in hospital. The identification of four vulnerability profiles provides insight into where best practice interventions can be targeted to address preexisting physical difficulties.

This study has various limitations. We acknowledge that some measurement bias could have occurred because patients self-reported their function retrospectively, whereas objective performance based assessments have greater validity [37]. However, the VES-13 is a validated tool with established psychometric properties including predictive validity [20]. Furthermore, the data collector asked elders questions to confirm current in-hospital performance with their retrospective reports. We conducted the study at one site, thereby limiting the generalizability of findings. Ongoing comparisons investigating patient characteristics in six similar wards in four other metropolitan hospitals in Victoria, Australia will help establish the generalisability of study findings in the future.

## Conclusions

A high level of vulnerability to functional decline was identified in hospitalised older general medicine patients. Highest difficulty performing ADLs was identified in those aged 85 years and over. Our findings support the need for those involved in health service planning, delivery and education to emphasise provision of 24-hour care that addresses vulnerability to functional decline in older general medicine patients. We identified four patterns of physical impairment associated with vulnerability to functional decline, supporting the need to deliver individualised care. The question of which class a new patient would belong to requires additional clinical investigation. This could be addressed in future research. Despite local government initiatives to guide practice [38] the problem of functional decline in hospitalized elders persists [6], suggesting a need for further investigation into the system and processes of 24-hour care provided to minimise functional decline in vulnerable elders in the Australian context.

## Abbreviations

ADL, activities of daily living; ICD-10, international classification of diseases 10 – 2010 Version; VES-13, vulnerable elders survey
